# Intracorporeal Heat Distribution from Fully Implantable Energy Sources for Mechanical Circulatory Support: A Computational Proof-of-Concept Study

**DOI:** 10.3389/fbioe.2017.00060

**Published:** 2017-10-17

**Authors:** Jacopo Biasetti, Aliaksei Pustavoitau, Pier Giorgio Spazzini

**Affiliations:** ^1^Department of Mechanical Engineering, Johns Hopkins University, Baltimore, MD, United States; ^2^Department of Anesthesiology and Critical Care Medicine, Johns Hopkins Medicine, Baltimore, MD, United States; ^3^Mechanics Division, National Institute of Metrological Research, Turin, Italy

**Keywords:** heat transfer, waste heat, mechanical circulatory support, nuclear power source, blood flow, aorta, intracorporeal heat distribution

## Abstract

Mechanical circulatory support devices, such as total artificial hearts and left ventricular assist devices, rely on external energy sources for their continuous operation. Clinically approved power supplies rely on percutaneous cables connecting an external energy source to the implanted device with the associated risk of infections. One alternative, investigated in the 70s and 80s, employs a fully implanted nuclear power source. The heat generated by the nuclear decay can be converted into electricity to power circulatory support devices. Due to the low conversion efficiencies, substantial levels of waste heat are generated and must be dissipated to avoid tissue damage, heat stroke, and death. The present work computationally evaluates the ability of the blood flow in the descending aorta to remove the locally generated waste heat for subsequent full-body distribution and dissipation, with the specific aim of investigating methods for containment of local peak temperatures within physiologically acceptable limits. To this aim, coupled fluid–solid heat transfer computational models of the blood flow in the human aorta and different heat exchanger architectures are developed. Particle tracking is used to evaluate temperature histories of cells passing through the heat exchanger region. The use of the blood flow in the descending aorta as a heat sink proves to be a viable approach for the removal of waste heat loads. With the basic heat exchanger design, blood thermal boundary layer temperatures exceed 50°C, possibly damaging blood cells and proteins. Improved designs of the heat exchanger, with the addition of fins and heat guides, allow for drastically lower blood temperatures, possibly leading to a more biocompatible implant. The ability to maintain blood temperatures at biologically compatible levels will ultimately allow for the body-wise distribution, and subsequent dissipation, of heat loads with minimum effects on the human physiology.

## 1 Introduction

Mechanical circulatory support devices, such as total artificial hearts (TAHs) and left ventricular assist devices (LVADs), are currently powered by external energy sources connected through percutaneous cables, significantly affecting patients’ quality of life (MacIver and Ross, 2012). Transcutaneous energy transfer technology, not yet cleared for clinical use, relies on external and internal coils for inductive power transmission, therefore avoiding piercing the skin. Despite significant improvements compared to the use of percutaneous cables, this technology still present several drawbacks: for example, the potential for skin damage due to overheating, and misalignment of the coils with subsequent loss of power transmission. Moreover, untethered operation still relies on batteries located inside the patient’s body that must be recharged every 1–6 h (Knecht et al., 2014).^1^

During the past decades significant effort has been devoted to the development of a fully implantable energy source able to provide long-term power for TAHs and LVADs. In particular, the use of radioisotopes has received considerable attention (Huffman et al., 1974; Whalen et al., 1974; Poirier, 2012; Tchantchaleishvili et al., 2012). Reinforced capsules of *α*-emitting materials, such as plutonium 238 (^238^Pu), can in principle be employed as fully implantable thermal energy sources (Huffman et al., 1974; Tchantchaleishvili et al., 2012). The heat generated by the nuclear decay can then be either directly converted into electricity or being used to drive a vapor cycle. Due to the limited efficiency of the energy conversion process, a large amount of waste heat, estimated at over ≈60 W for TAH-rated energy sources, is generated and needs to be dissipated.

Failure in dissipating this additional heat load can result in heat stroke, as shown during animal trials (Huffman et al., 1974), leading to multi-organ injury and death (Bouchama and Knochel, 2002). Focal heat exposure from internal heat sources can also injure surrounding tissues, including muscle, blood vessels, and blood. Minimizing the effects of heat exposure and understanding critical exposure times to heat is mandatory for the design of heat exchangers for mechanical circulatory support devices. Generally, heat-induced alterations depend on blood/tissue temperature and duration of exposure. Table 1 summarizes heat exposure effects on tissues, cells, and proteins. As reported in Table 1, the most vulnerable cells are polymorphonuclear leukocytes and platelets, participating in the immune function and hemostasis, respectively. Thus, focusing on those functions during clinical implementation of studied devices is crucial.

**Table 1:** Effects of hyperthermia on molecules, cells, and tissues.

Molecules, cells, and tissues	Effect	Temperature and exposure time	Reference
Red blood cells	Hemolysis	50°C × 1 h = 6%	Utoh et al. (1992)^a^
		50°C × 7 h = 10%	Gershfeld and Murayama (1988)^a^
		45°C × 10 h or 40°C × 30 h = 1%

	Morphologic changes (spherocytosis, echinocytosis)	50°C × 1 h = 97%	Utoh et al. (1992)^a^

Polymorphonuclear leukocytes	Phagocytosis	44°C × 0.5 h = 33% phagocytosis (vs. 93% at 37°C)	Utoh and Harasaki (1992)^a^

Platelets	Decreased aggregation and adhesion with increased risk of bleeding	44°C × 1 h = 6.2% of baseline binding to GPIIb-IIIa receptor	Pasha et al. (1995)^a^
		44°C × 1 h = 2.1% maximum ADP-induced aggregability 43°C × 5 min = less than 10% maximum aggregability with any agonist	Choi and Pai (2002)^a^

	Thrombin-stimulated release of proteins from *α*-granules	42°C × 10 min = near complete absence of release	Etulain et al. (2011)^a^

Coagulation and fibrinolysis	Activation with resultant DIC	Observed in heatstroke patients with rectal temperature ≥42.3 ± 0.2°C	Bouchama et al. (1996)^b^

Blood proteins	Denaturation with loss of function	Temperatures greater than 45°C	Gershfeld and Murayama (1988)^a^

Endothelium	Direct damage with multi-organ dysfunction	Heatstroke patients with rectal temperature ≥41.2°C	Sohal et al. (1968)^c^
	
		In calves implanted with a 50 W energy source	Huffman et al. (1974)^b,c^

Surrounding tissues	Heat-induced angiogenesis	Observed in calves under a temperature increase of T = 4.5 ± 0.2°C above body temperature after 2 weeks	Davies et al. (1994)^b^
	
	Muscle necrosis and capsule formation	Necrosis in calves at tissue interface temperature T = 45.3 ± 2.2°C; and after 7 weeks capsule formation with temperature lowering to 41.8 ± 0.5°C	Seese et al. (1998)^b^

*^a^In vitro*.

*^b^In vivo*.

*^c^Ex vivo*.

*DIC, disseminated intravascular coagulation*.

Animal studies have investigated the effect of heat on animal’s physiology generated by ^238^Pu heat sources with thermal output of 16 and 24 watts (W) in calves, and electric heaters with output powers up to 50 W in dogs (Huffman et al., 1974). In these studies, the blood flow in the descending aorta was used as heat sink to distribute the heat generated locally throughout the animal’s body. Over 2-year survival was reported despite a three-fold increase in the mean respiratory rate, suggesting a possible thermoregulatory mechanism for coping with the extra intracorporeal heat (Whalen et al., 1974).

The success of this technology for clinical use depends, to a great extent, on designs that avoid exposing blood and the surrounding tissues to supra-physiological temperatures. Therefore, the aim of the present paper is to evaluate the heat-exchanging capabilities of different heat exchanger designs under heat loads of 64 W, for TAHs, and 24 W, needed to power an LVAD in humans (Poirier, 2012; Wang et al., 2014). In particular, blood temperature distributions at the exchanging surfaces, in the bulk, and blood cells’ temperature histories are analyzed. The work shows that with carefully designed heat exchangers the extra heat loads can be intracorporeally distributed with limited exposure to supra-physiological temperatures. Further refinements of the heat exchanger design should allow for temperatures to be fully contained in the physiological range.

## 2 Materials and Methods

### 2.1 Intracorporeal Heat Distribution

**Figure 1:**
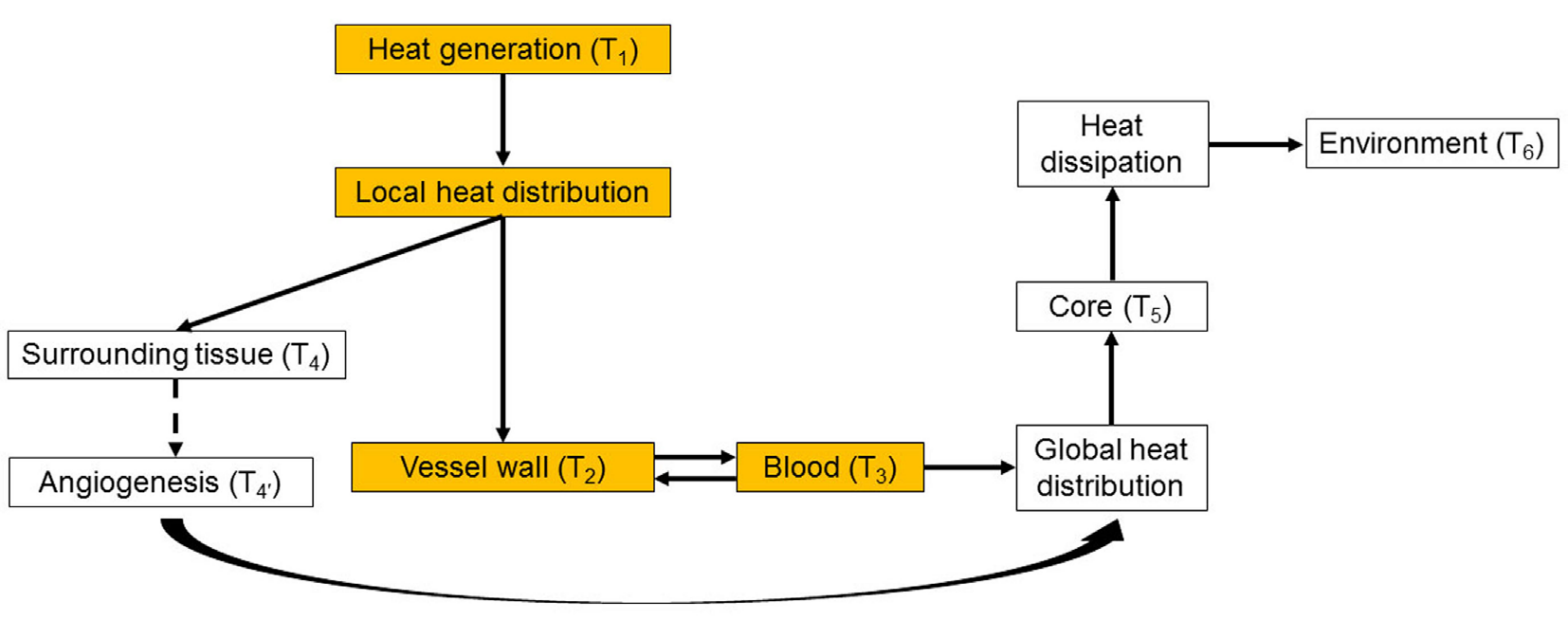
Heat distribution and dissipation pathways from the heat source to the environment. The colored boxes represent the intracorporeal distribution step, which is the focus of the present work. While in this work the generated heat is transferred directly to the blood without passing through the vessel wall, in the general case the vessel wall can be part of the heat-exchanging pathway. See main text for details.

Intracorporeal heat distribution and extracorporeal dissipation pathways from the heat source to the environment are schematically presented in Figure 1. This work focuses on the heat distribution step, i.e., on how the waste heat generated by the power source is transmitted to, and distributed into the blood stream (colored boxes in Figure 1). In this diagram, the power generation system releases waste heat, at temperature T_1_, which is then distributed locally through direct contact to the surrounding tissues (predominantly muscle), with resulting temperature T_4_, vascular walls, T_2_, and blood, T_3_. Blood distributes the waste heat throughout the body, increasing the core temperature to T_5_ > 37°C, until it is dissipated to the environment, with temperature T_6_. Long-term heat exposure results in angiogenesis within tissues directly exposed to excessive heat, with resultant decrease in temperature from T_4_ to ${\text{T}}_{4}^{\prime }$ due to enhanced heat removal achieved by the increased local blood flow (Emoto et al., 1988; Davies et al., 1994; Seese et al., 1998).

### 2.2 Vascular Geometry and Blood Flow Modeling

Idealized rigid-wall vascular geometries accounting for the ascending aorta, aortic arch, and the descending/abdominal aorta have been modeled based on *in vivo* data from Alastruey et al. (2016). The brachiocephalic, common carotid, and subclavian branches have also been included, see Figure 3 and Table 2. Heat exchangers have been inserted by replacing a segment of the descending aorta, refer to Section 2.3 for details.

**Table 2:** Idealized vascular geometry dimensions.

Ascending aorta	Aortic arch	Descending/abdominal aorta	Brachiocephalic artery	Common carotid artery	Subclavian artery
*∅*: 2.5 cm	*∅*: 2.5 cm	*∅*: 2.5–2.0 cm	Inlet *∅*: 1.4 cm	Inlet ∅: 1.02 cm	Inlet ∅: 1.06 cm
–	Radius of curvature: 5.1 cm	Length: 23 cm	Outlet *∅*: 0.86 cm	Outlet *∅*: 0.58 cm	Outlet *∅*: 0.68 cm
–	–	–	Length: 4.20 cm	Length: 3.23 cm	Length: 3.31 cm

*∅: diameter*.

**Figure 2:**
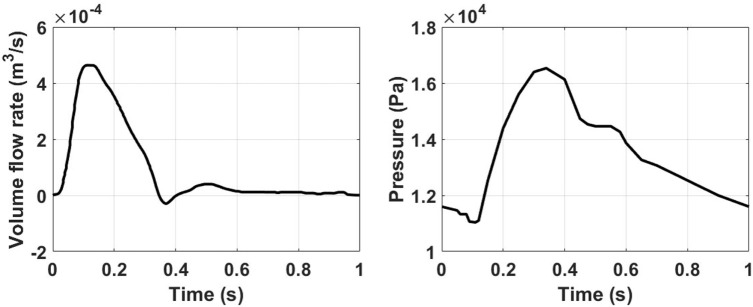
Ascending aorta inlet volume flow rate (left) obtained from Alastruey et al. (2016), and outlet pressure waveforms (right) taken from Biasetti et al. (2010, 2011).

Physiological volume flow rates have been applied at the ascending aorta inlet, and at the brachiocephalic, common carotid, and subclavian outlet branches. A pressure waveform has been imposed at the descending aorta outlet. The ascending aorta waveform has been derived from Alastruey et al. (2016), while the ones for the secondary branches have been created by scaling the inlet waveform according to the outlet flow rates provided in Alastruey et al. (2016). The outlet pressure waveform has been derived from Biasetti et al. (2010, 2011). The inlet and outlet waveforms are reported in Figure 2. Since this model considers rigid walls and incompressible fluid, the pressure waveform effect is to ensure physiological absolute pressure values.

Blood has been modeled as an incompressible, non-Newtonian shear-thinning fluid with constant density and its shear-thinning behavior modeled by the Carreau–Yasuda constitutive model (Biasetti et al., 2011). Blood constitutive parameters are reported in Table 3.

**Table 3:** Blood density, *ρ*_b_, and Carreau–Yasuda parameters: μ_∞_ and μ_0_ represent blood viscosity at the upper and lower ends of the shear rate spectrum; while *λ*, n, and a control the transition between these extreme conditions.

*ρ*_b_ (kg/m^3^)	μ_∞_ (Pa s)	μ_0_ (Pa s)	*λ* (s)	n	a
1,050	0.0035	0.16	8.2	0.2128	0.64

### 2.3 Heat Source and Heat Exchanger Models

**Figure 3:**
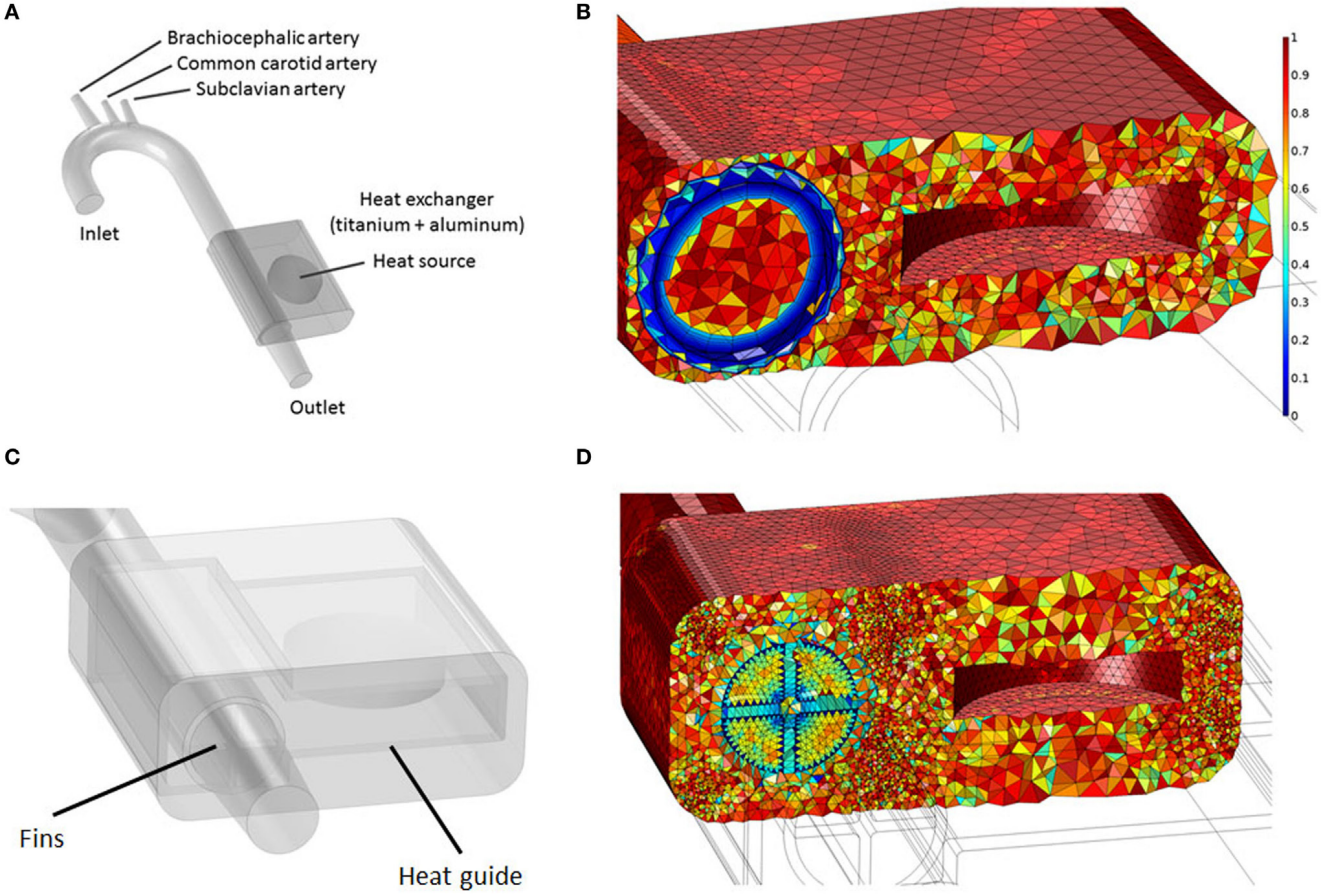
**(A)** Idealized aortic geometry with the 64 W heat source and heat exchanger’s base design connected to the descending aorta; **(B)** Aorta, heat source internal surface, heat conductor, and heat exchanger section showing the finite element mesh. The color scale indicates element quality; boundary layer elements present a lower quality due to their higher aspect ratio; **(C)** 64 W with fins and heat guide design; **(D)** Cross section showing the finite element mesh for the 64 W with fins and heat guide design.

Two waste heat output levels have been considered: (i) 64 W, simulating a TAH-rated power source and (ii) 24 W, mimicking an LVAD-rated one. In all cases, following the original design used in animal tests conducted by Huffman et al. (1974), the power sources, located on the left side of the descending aorta, are embedded in a conductive housing made of aluminum. Blood flows in a 10-cm-long titanium conduit, the heat exchanger, embedded in the main aluminum body, see Figure 3 and Table 5. This design is referred from now on as “base design.” Two additional designs aimed at enhancing the heat transfer are modeled. The first one considers the addition of two longitudinal titanium fins, perpendicular to each other, with a thickness of 2 mm, spanning the diameter of the flow channel and the entire length of the heat exchanger, while the second one takes into consideration, in addition to the fins, a copper heat guide. This guide consists of a 0.25-cm-thick sheet of copper wrapped around the heat source and the blood conduit and extending through the heat-exchanging region, with the exception of the first and last 1 cm regions so to have a fully contained heat guide. The copper guide is positioned 0.25 cm away from the surface of the heat source and 0.15 cm from the luminal surface, see Figure 3 for details. The role of the copper heat guide is to conduct heat toward the far side of the aluminum casing to achieve a more homogeneous temperature distribution in the metal before transferring heat to the blood. The choice of titanium as the blood-contacting material was made to (i) be as consistent as possible with the heat exchanger used in Huffman et al. (1974) and (ii) for its biocompatibility characteristics (Jamiolkowski et al., 2015, 2016). The heat source has been modeled as an internal cylindrical surface ejecting a total output of 64 or 24 W. Thermal material properties are reported in Table 4.

**Table 4:** Heat capacity and thermal conductivity data for aluminum, titanium, copper, and blood.

	Aluminum	Titanium	Copper	Blood
Heat capacity J/(kg⋅K)	900	710	385	3,650
Thermal conductivity W/(m⋅K)	238	7.5	400	0.5

**Table 5:** Heat conductor and heat source dimensions.

	HC streamwise length (cm)	HC width (cm)	HC thickness (cm)	HS diameter (cm)	HS height (cm)	HS volume (cm^3^)
24 W (b)	10	7.8	3.1	2.8	1	6.16
64 W (b,f)	10	9	3.1	4.6	1	16.62
64 W (fhg)	10	10.5	4.3	4.6	1	16.62

*b, base design; f, fins design; fhg, fins with heat guide design*.

### 2.4 Modeling Equations, Finite Element Discretization, and Solver

A computational model of blood flow coupled with a fluid–solid heat transfer model has been implemented. For the fluid domain, the incompressible continuity [equation (1)], momentum [equation (2)], and energy equations [equation (3)] have been solved:

(1) ∇⋅u=0

(2) $$\rho_{\text{b}}\frac{\partial u}{\partial \text{t}}+\rho_{\text{b}}(u\cdot \nabla )u=\nabla \cdot \left(-p\text{I}+\mu_{\text{b}}\left(\nabla u+{\left(\nabla u\right)}^{\text{T}}\right)\right)$$

(3) $$\rho_{\text{i}}C_{{\text{p}}_{\text{i}}}\frac{\partial \text{T}}{\partial \text{t}}+\rho_{\text{i}}C_{{\text{p}}_{\text{i}}}u\cdot \nabla \text{T}+\nabla \cdot \left(-k_{\text{i}}\nabla \text{T}\right)=0$$

For the solid domain, only the energy equation without the convective contribution *ρ*_i_*C*_pi_**u**⋅∇T has been solved. The surface heat source boundary condition has been implemented as a total heat flux modeled as − **n**⋅**q** = P_0_/A, where **n** is the surface normal, **q** the surface total heat flux in W/m^2^, P_0_ the total heat output in W, and A the surface area of the heat source. In the above equations, **u** is the blood velocity vector in m/s, *p* the blood pressure field in Pa, *ρ*_i_ the density in kg/m^3^, $C_{{\text{p}}_{\text{i}}}$ the specific heat at constant pressure in J/(kg⋅K), and *k*_i_ the thermal conductivity of the i-th material in W/(m⋅K).

The velocity, pressure and temperature fields have been discretized with Lagrangian linear elements. A tetrahedral dominated mesh has been employed throughout the fluid and solid domains, with the addition of eight layers boundary layer mesh throughout the fluid domain in the base design and in the aortic tract proximal to the heat exchanger in the fins and fins with heat guide designs, see Figure 3 and Table 6 for details. Swept meshing has been employed wherever possible to reduce the computational cost. A convergence study has been conducted by incrementally increasing the number of elements in the fluid and solid domains until no appreciable differences in velocity and temperature distributions, at selected locations throughout the domains, were observed. Due to the long thermal transient from the uniform initial condition of 37°C to the final periodic solution, a two-step approach has been employed. First, a steady-state solution, using average inlet and outlet waveform values, has been calculated. From this solution, a transient analysis has been performed for a number of cardiac cycles sufficient to achieve periodicity (between 5 and 10 cycles depending on the case). Both the steady and transient analysis employed a two-step segregated solver, with the first step solving for the velocity and pressure while the second for the temperature field. Both steps employed a direct PARDISO solver. An adaptive BDF scheme, with a maximum order of two, and adaptive time stepping has been employed for time integration. The adaptive time step varies between 10^−8^ and 10^−2^ s, with data saved in output every 10^−2^ s. Computations have been performed using COMSOL Multiphysics 5.2a on a 64-bit Windows 7 machine with four Intel^®^ Core™i7-4790K CPU at 4.00 GHz, 32GB of RAM.

**Table 6:** Mesh details for the different analyzed cases and designs.

	24 W base	64 W base	64 W fins	64 W fins with heat guides
Total number of elements	211,923	262,060	366,424	1,177,388
Total number of DoF	288,352	302,349	364,071	531,230

*The more complex flow field induced by the presence of the fins required a higher number of elements in the fluid domain. The even higher number of elements in the fins with heat guide design is a consequence of the larger physical dimension of the model and also of the element concentration around the corners of the heat guide*.

### 2.5 Cell Tracking

To evaluate the temperature time-history of platelets and red blood cells as they flow through the heat-exchanging region, a particle tracking analysis has been conducted. Cell’s density has been assumed to be *ρ_p_* = 1,050 kg/m^3^ for all cases, while their diameter has been set to d*_plt_* = 3μm for platelets, and d*_rbc_* = 7μm for red blood cells (Mezzano et al., 1981; Polanowska-Grabowska et al., 1992; Diez-Silva et al., 2010). Cells have been seeded at the inlet of the heat-exchanging section and tracked for 10 cardiac cycles, discarding the first one due to spurious initialization effects. The seeding distribution for platelets over the cross section has been set proportional to the third power of the radial location, maximum at the wall and minimum at the center, as platelets are predominantly located near the wall (Aarts et al., 1988). Red blood cells were instead distributed uniformly over the cross section. Particle motion is governed by Newton’s Law:

(4) $$\frac{d(m_{p}v)}{\mathrm{dt}}=F_{\text{t}}$$

where **v** is the particle velocity and the term **F**_t_ represents the drag force acting on each particle. The drag force has been defined as:

(5) $$F_{\text{t}}=\frac{1}{\tau_{p}}m_{p}\left(u-v\right)$$

where

(6) $$\tau_{p}=\frac{\rho_{p}d_{p}^{2}}{18\mu_{b}}$$

is the particle response time. When a cell reaches an outlet surface its tracking stops, while when it collides with the arterial or heat exchanger surfaces it specularly reflects so that its momentum is conserved. To achieve this, the cell’s post-collision velocity is defined as **v** = **v**_c_ − 2(**n**⋅**v**_c_)**n**, where **v**_c_ is the cell velocity at the moment of impact with the solid surface and **n** the surface’s normal vector.

### 2.6 Thermal Exposure Index—TEI

To quantify the heat load experienced by the cells a *thermal exposure index*, or TEI, has been defined as follows:

(7) $$\text{TEI}=\sum_{i=1}^{n-1}\left(\Delta {\text{t}}_{\text{i}}\cdot \frac{\left({\text{T}}_{\text{i}+1}+{\text{T}}_{\text{i}}\right)}{2}\right)$$

where *n* is the total number of saved time data points. This parameter takes into account both the temperature cells are subjected to and the respective exposure times.

## 3 Results

### 3.1 Baseline Design, 64, and 24 W Cases

**Figure 4:**
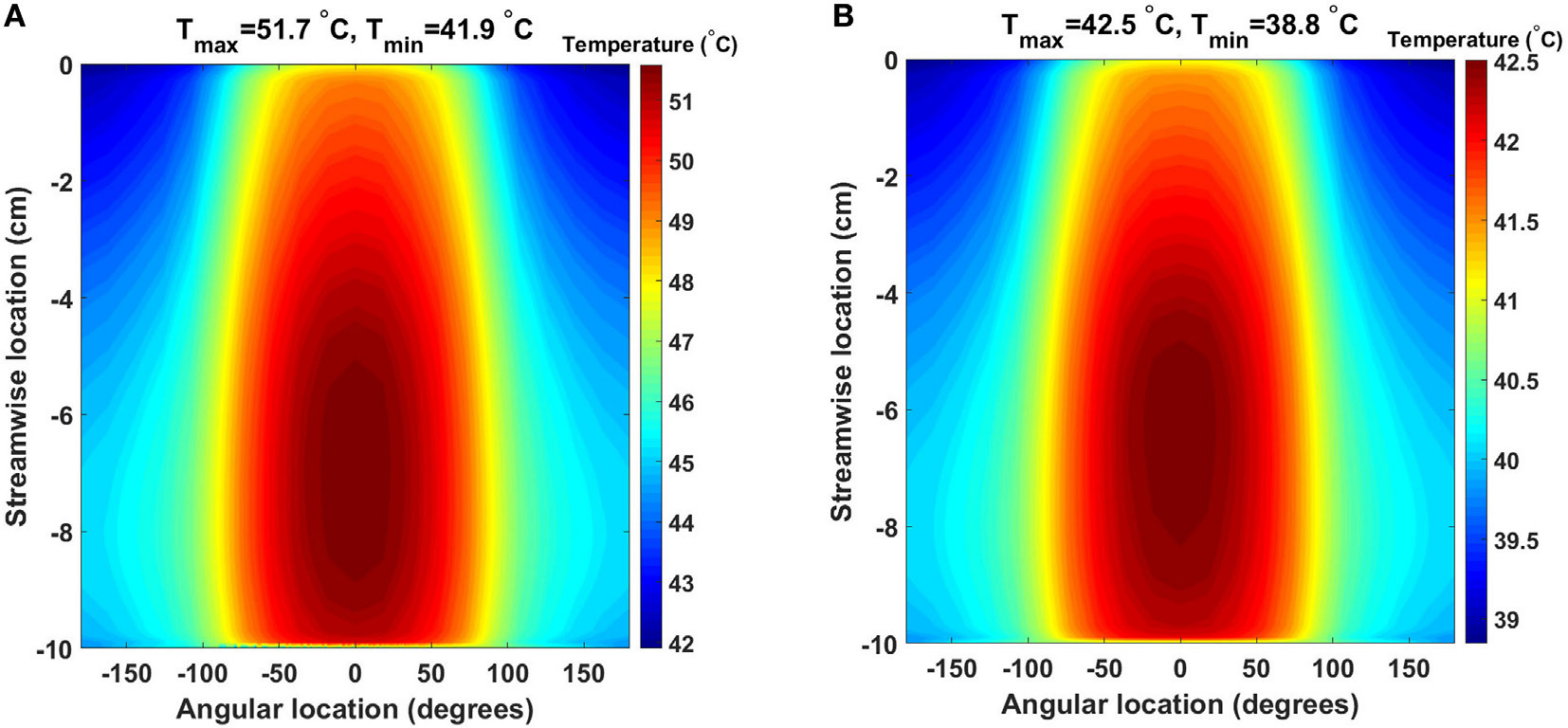
Time-averaged temperature distribution over five cardiac cycles at the heat exchanger interface with the flowing blood in the **(A)** 64 W and **(B)** 24 W base cases, respectively. The color scale is different for the two plots, and the original cylindrical surface of the heat exchanger has been mapped onto a plane for easier visualization. The x-axis indicates the angular direction (±180°), while the y-axis indicates the streamwise direction with y = 0 cm representing the inlet and y = −10 cm the outlet (blood flow from top to bottom). Peak temperatures are located on the side closer to the heat source.

The baseline design, for the 64 and 24 W cases, presents very similar temperature and heat flux patterns, both in the solid heat conductor/exchanger and in the blood flow, with the main difference being the temperature range. In Figure 4, the temperature distribution, averaged over five cardiac cycles, at the heat exchanger interface with blood for the two cases is presented. In this plot, the cylindrical surface of the heat exchanger has been mapped to a plane, with different color scales for the two cases for clearer visualization. The zero angular location lays on the model’s symmetry plane, on the side closer to the heat source. As expected, the temperature distribution indicates that the side closer to the heat source and, therefore, the blood flowing closer to it, receives a substantially higher heat load compared to the opposite side. Moreover, peak temperatures are located at 70% and approximately 60% of the heat exchanger stream wise length (50% marking the center of the heat source) in the 64 and 24 W cases, respectively, due to the different geometrical sizes of the heat sources. The downstream shift with respect to the 50% position is due to the convective nature of the heat exchange. As Figure 4 indicates, blood receives a thermal load that increases with the streamwise coordinate, meaning that cells and proteins are exposed to an increasing thermal load as they move through the heat exchanger, see Section 3.3. Both models present surface temperatures much higher than the physiological blood temperature of 37°C and well into the range known to cause protein denaturation, cell damage, and coagulation activation (Gershfeld and Murayama, 1988; Bouchama et al., 1996). Moreover, these temperature levels are not compatible with the formation of an endothelial layer, therefore impacting the biocompatibility of the implant (Huffman et al., 1974). At the distal end of the heat exchanger, a steep temperature gradient is observed. This layer is the result of (i) the sudden termination of the heat-exchanging surface and (ii) the applied insulating boundary conditions in the rest of the domain, see Section 4.

**Figure 5:**
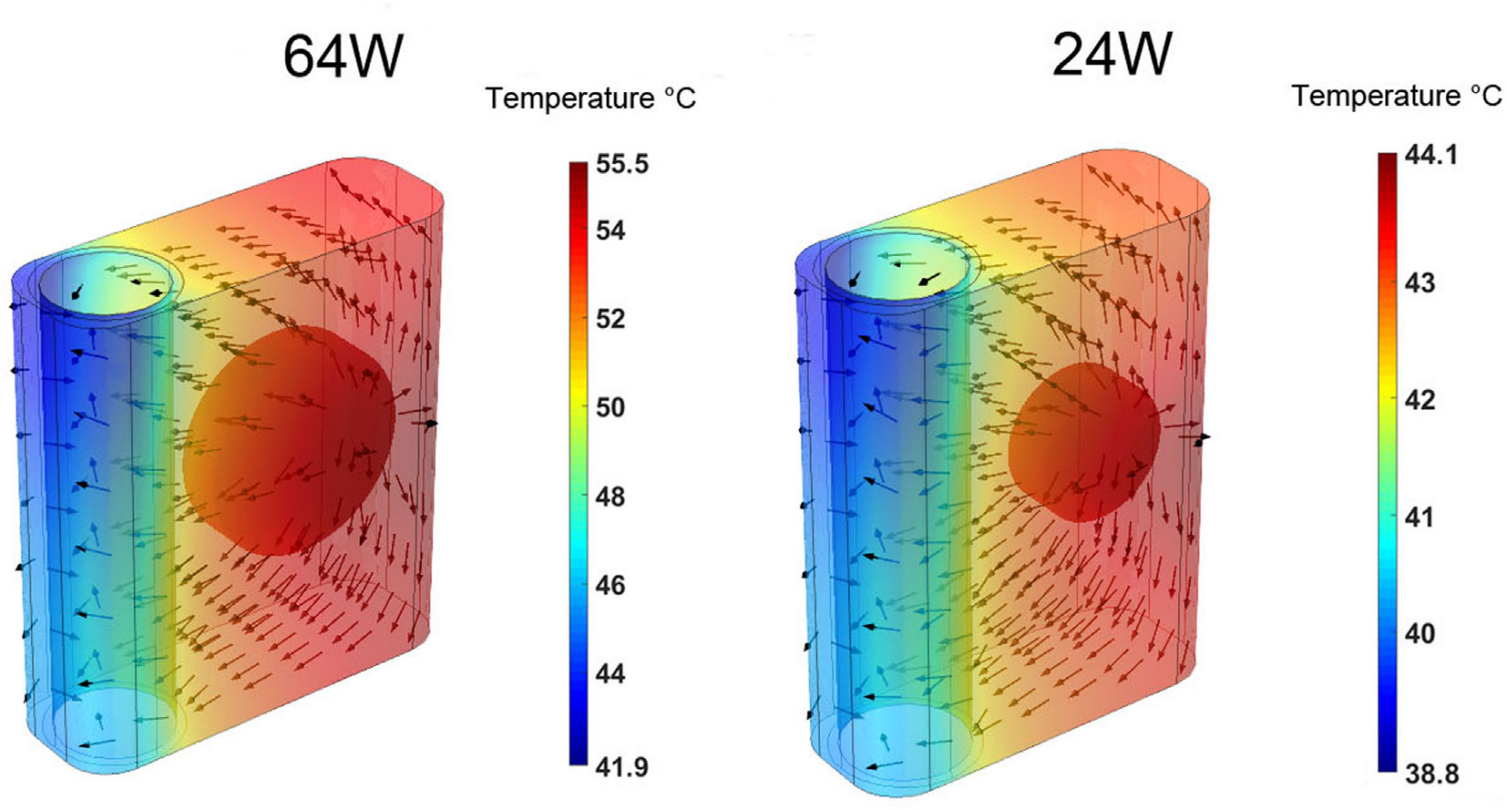
Time-averaged over five cardiac cycles temperature contours and heat flux vectors in the heat conductor/exchanger assembly for the (left) 64 W base case and for the (right) 24 W base one. The color scale is different for the two cases.

The time-averaged, over five cardiac cycles, temperature and heat flux distributions in the heat conductor/exchanger assembly for the 64 and 24 W base cases are shown in Figure 5. In both cases, the highest temperatures are located near the heat source on the side opposite to the blood flow, while the lowest ones are found near the blood domain on the side opposite to the heat source. The heat flux vectors show how the energy flow is directed toward the heat sink realized by the flowing blood. Most of the heat exchange with the blood happens on the side closer to the source, and only a minor part is realized on the far side, explaining the results presented in Figure 4. Note that, in this paper, perfect insulation at the external boundaries of the heat conductor is assumed. This, in turn, implies that the entire heat load generated by the source is transferred to the blood flowing in the aorta. In reality, a fraction of the heat would be transmitted to the surrounding tissues, see Section 4. The results indicate that the majority of the heat is exchanged with the blood through a limited region of the heat exchanger, namely through the surface closer to the heat source. This heat flux concentration on a relatively small surface, in conjunction with the stable flow velocity profile, explains the elevated local temperatures observed.

**Figure 6:**
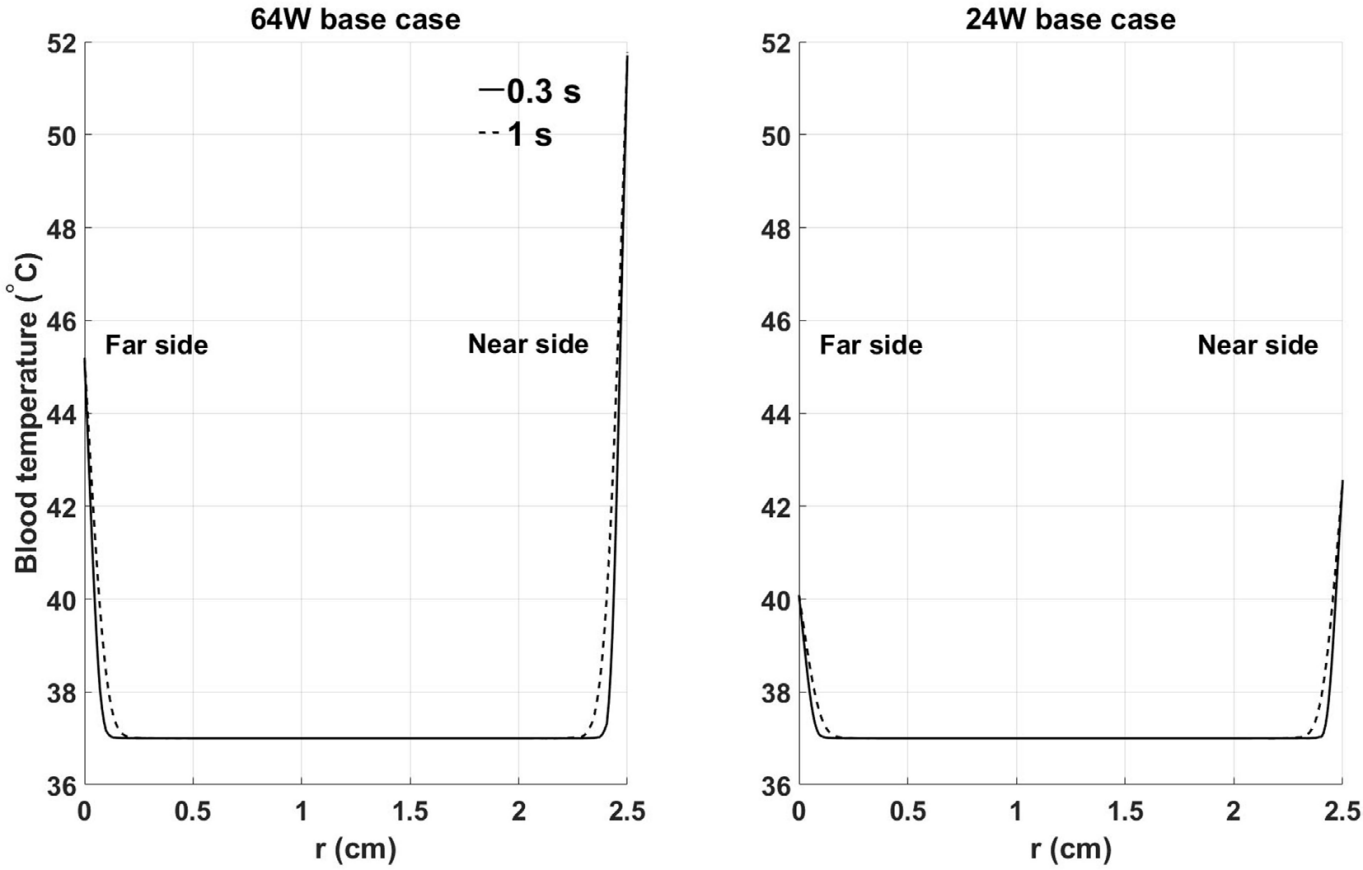
Instantaneous temperature distribution along a cross-sectional cut line, inside the blood domain, located at 70% of the streamwise length of the heat exchanger: (left) 64 W base case, (right) 24 W base case. Far and near side refer to the sides opposite and adjacent to the heat source, respectively. Time points have been chosen to show the thinnest (0.3 s) and thickest (1 s) thermal boundary layer profiles. X-axis coordinate r spans the entire diameter of the aortic cross section.

To characterize the temperature distribution inside the blood stream, Figure 6 shows the instantaneous temperature distribution along a cross-sectional cut line, inside the blood domain, located at 70% of the stream wise length of the heat exchanger. Two time points are reported: 0.3 s and 1 s, corresponding to the times when the thinnest and thickest thermal boundary layer profiles are observed. At both times, a sharp increase in blood temperature across the thermal boundary layer can be observed. As expected, the thermal boundary layer on the side closer to the heat source is characterized by higher temperatures. When considering that flow velocity in the boundary layer is lower than in the core of the flow, leading to a higher residence time of the blood particles, these data show that the thermal boundary layer is a potentially critical flow region for the survival of blood cells and proteins.

### 3.2 64 W Base, Fins, and Fins with Heat Guide Designs

**Figure 7:**
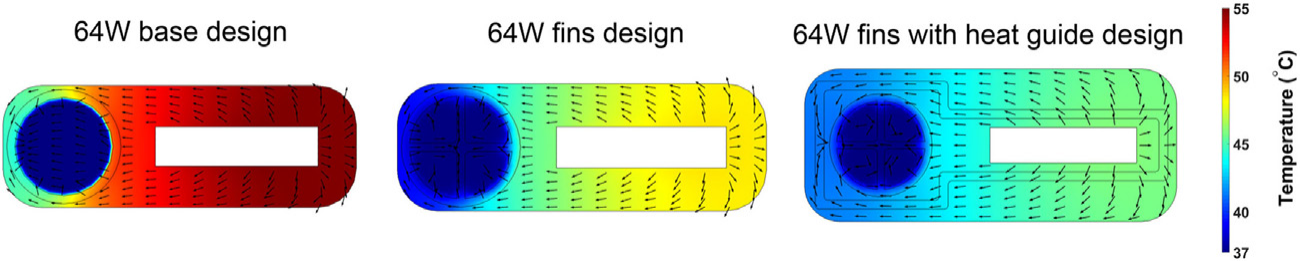
Time-averaged over five cardiac cycles temperature contours and heat flux vectors in the heat conductor/exchanger assembly and in inside the blood stream. The three 64 W designs are considered: (left) base design; (center) fins design; and (right) fins with heat guide design. The addition of heat transfer/guide elements lowers the maximum temperature and allows for a more homogeneous heat distribution.

The 64 W case is the most demanding from a heat distribution and dissipation standpoint. Therefore, the analysis of different heat-exchanging geometries will be based on this case. The time-averaged, over five cardiac cycles, temperature distribution and heat flux vectors in one cross section located in the center of the heat conductor/exchanger for the three 64 W designs are shown in Figure 7. The data show how the progressive addition of heat transfer/guide elements enhances and spatially homogenizes the transfer of thermal energy from the heat source to the blood resulting in a substantial decrease in the maximum temperature, and in a more homogeneous temperature distribution in the solid and in the blood. In particular, the direction of the heat flux vectors inside blood changes from being predominantly directed from the near side to the far side in the base design, to being randomly oriented in the fins with heat guide design, an indication of the disappearance of the strong intra-blood temperature gradient present in the base design.

**Figure 8:**
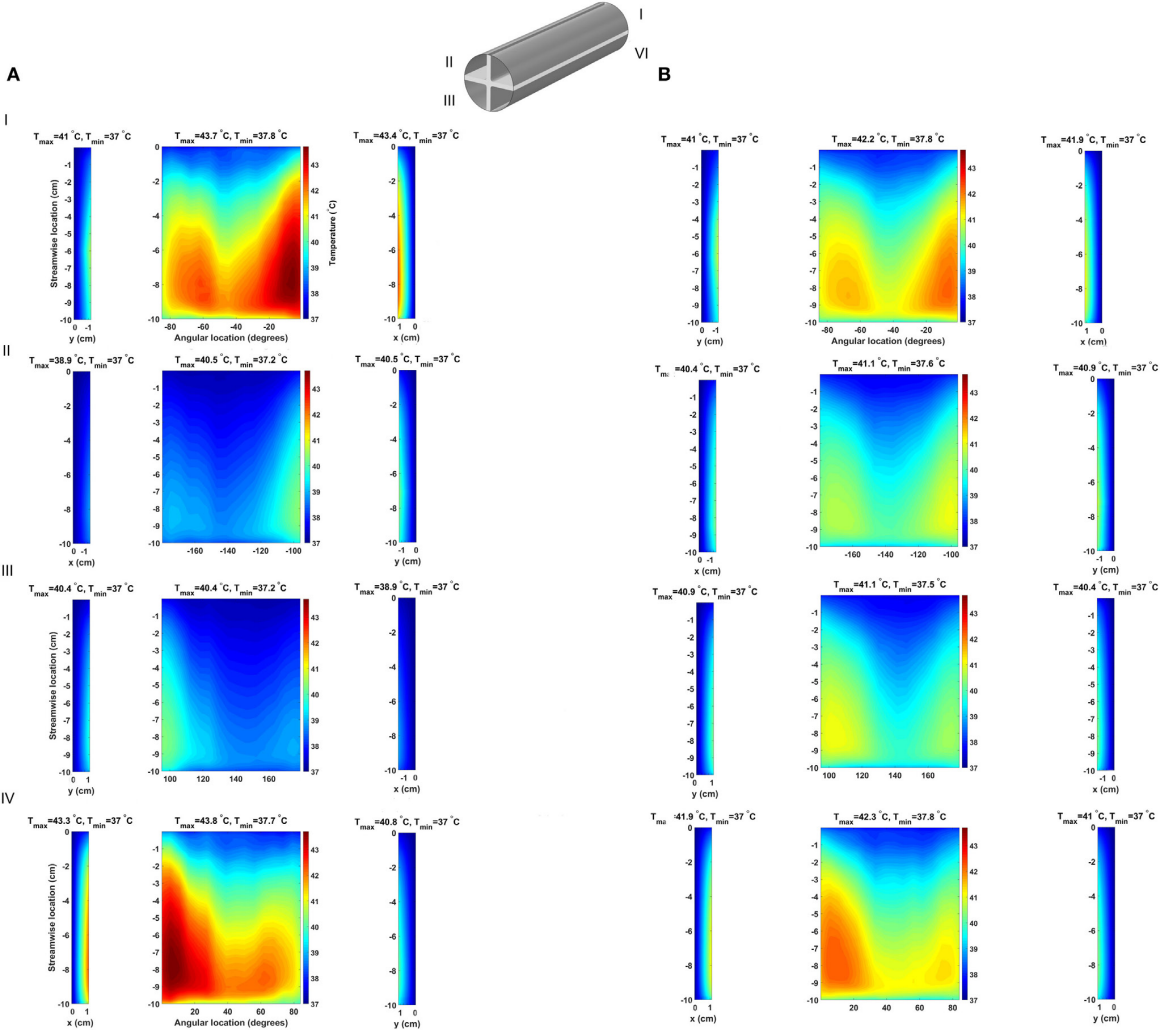
Time-averaged temperature over five cardiac cycles at the blood–heat exchanger interface in the **(A)** fins design and **(B)** fins with heat guide design. The latter achieves a lower maximum temperature compared to the former and, perhaps more important, a more uniform temperature distribution across the blood-contacting surfaces. The x-coordinate indicates horizontal surfaces while the y-coordinate indicates the vertical ones.

The consequences of altering the heat exchanger geometry are clearly illustrated in Figure 8, where the time-averaged, over five cardiac cycles, temperature distribution at the blood–heat exchanger interface in the 64 W case with fins and fins with heat guide are presented. To provide a clear visualization of the interface temperatures, each of the four flow-path conduits created by the fins is reported separately (I–IV), with the curved surfaces in the center and the horizontal and vertical ones on the sides. This figure should be compared with Figure 4, which reports the same data for the baseline case. As the temperature distribution indicates, the presence of the intraluminal fins, and of the heat guide, allows for the transmission of 64 W with substantially lower surface temperatures compared to base design, hence potentially enabling less cell and protein damage, and the formation of a viable endothelial layer. Of particular importance is the fact that the fins with heat guide design is able to transmit 64 W with peak temperatures lower than in the 24 W base design. The heat exchange process is largely modified because of (i) an increase in the useful surface for heat exchange; and (ii) because the perturbation to the flow field introduced by the presence of the fins increases mixing and, thus, the energy exchange within the boundary layer, leading to a reduction of the peak temperature. By comparing the two designs, fins and fins with heat guide, it is possible to observe that the qualitative behavior is very similar, this being a result of the flow field being equal. On the other hand, the addition of a heat guide helps in routing heat to the far side of the aortic section further reducing peak temperatures by allowing a larger portion of the thermal energy to be exchanged with the blood flowing on the far side of the aortic section. In addition, in the presence of heat conduction to the adjacent tissues, the fins with heat guide design case would require a much lighter thermal insulation compared to the base design, this due to the lower heat conductor temperatures involved.

**Figure 9:**
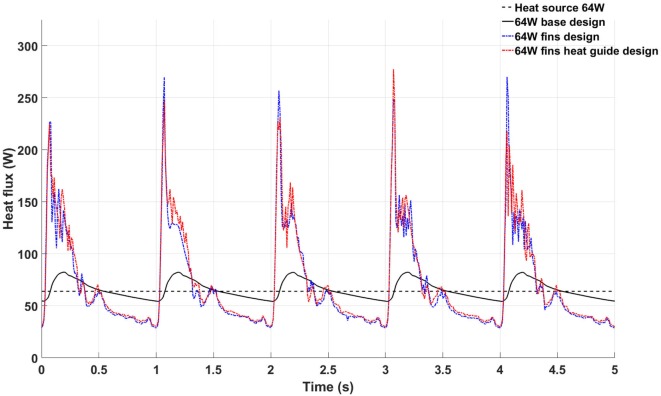
Temporal behavior of the total heat flux flowing inside the blood stream in the 64 W base (black continuous line), fins (blue continuous line), and fins with heat guide (red continuous line) designs. The black dashed line represents the power output of the heat source.

An informative parameter for the evaluation of the heat transfer capabilities of a given heat exchanger design is the temporal behavior of the total heat flux flowing inside the blood, see Figure 9. The 64 W base design shows heat flux values higher then the heat source output during systole and early diastole due to the enhanced convective heat transfer brought by the higher blood flow rate, while presenting lower values during late diastole due to the lower blood velocity and consequent lower convective heat transfer. Moreover, the heat flux shows a remarkable periodical behavior—a further indication of the convergence of the computational results. The heat flux in the 64 W fins and fins with heat guide designs are characterized by higher peak heat fluxes, also during peak systole, and a small degree of aperiodicity, with the presence of high frequency, small amplitude variations throughout the cardiac cycle. This behavior is related to the more complex flow field established by the presence of the fins in flow path: stagnation points and vortex shedding influence energy transmission. In particular, the peak heat flux achieved during systole is a result of the increased convective heat transfer, in turn caused by a more pronounced mixing and, possibly, turbulence. This leads to a higher waste heat removal without resulting in a pronounced increase in blood temperature due to the enhanced mixing.

**Figure 10:**
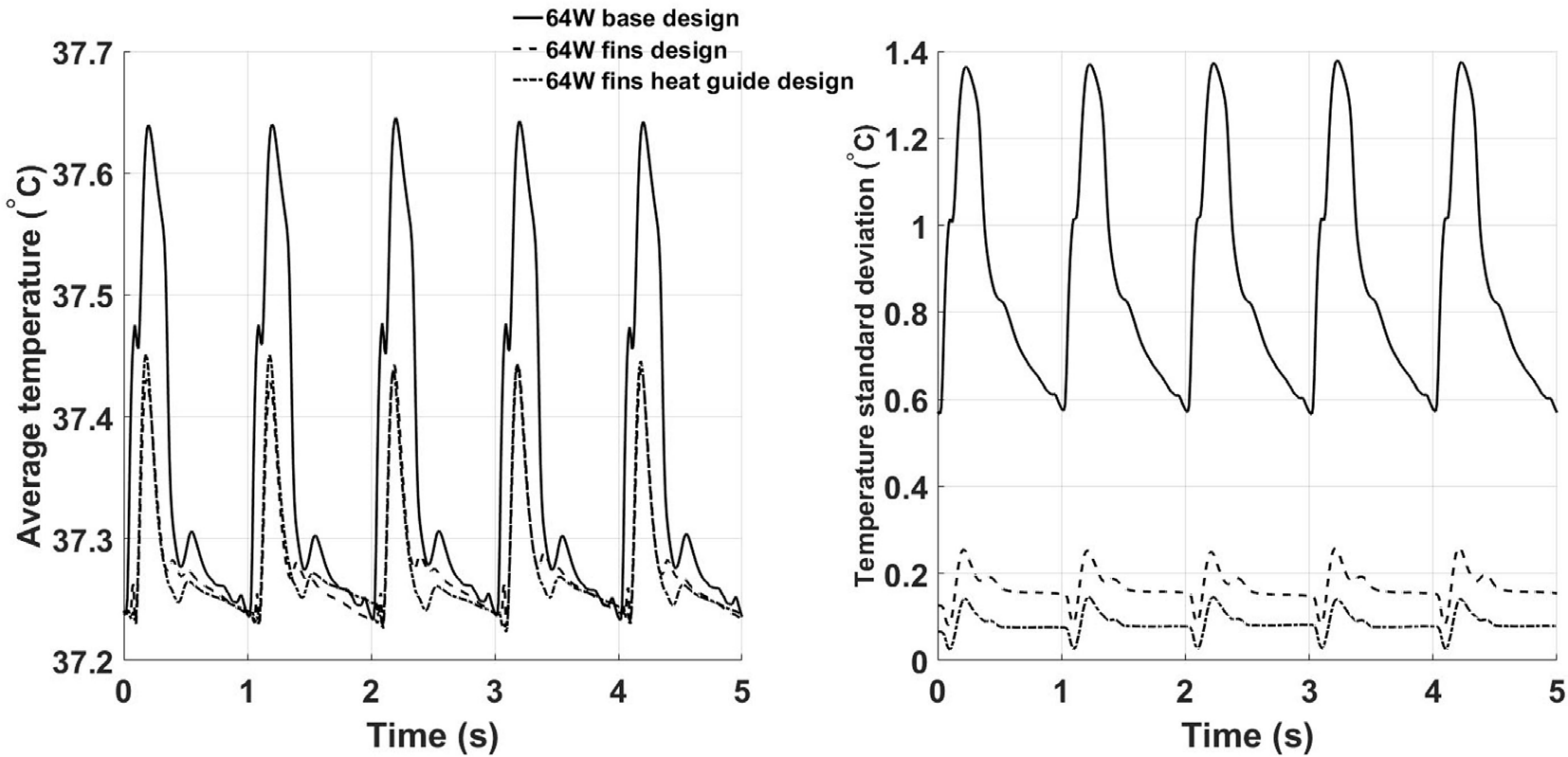
Time behavior of the space averaged temperature (left) and SD (right) calculated on the outlet plane for the three 64 W designs. The fins with heat guide design leads to the lowest average temperatures and SDs throughout the cardiac cycle.

To characterize the degree of mixing and, therefore, the ability to reach more uniform temperature distributions potentially less damaging to the blood components, the spatial average temperature and spatial standard deviation (SD) on the outlet surface have been calculated for the three designs, see Figure 10. The spatial average temperature in the base design case presents peak temperatures about 0.2°C higher than the fins and fins with heat guide design. This relatively small temperature increase is particularly significant considering that the core flow is mostly at 37°C, hence meaning that much higher temperatures are achieved in the thermal boundary layer of the base design case. Note that the overall lower temperatures in the fins and fins with heat guide designs are still consistent with the total power output of 64 W being removed by the blood flow, as this temperature is not a flux-weighted one but is the true average temperature felt by the cells flowing through the cross section. Most notably, the temperature SD for the fins and fins with heat guide designs is one order of magnitude lower than the one for the basic design case, indicating the achievement of the desired increased temperature uniformity across the cross section. In particular, the addition of the heat guide results in the lowest SD and, therefore, in the design that reaches the best temperature uniformity. Of note, the SD peaks, in all designs, right after peak systole and decreases throughout the rest of the cardiac cycle—an indication of the enhanced mixing brought about by the systolic mass flow.

### 3.3 Cell Tracking

**Figure 11:**
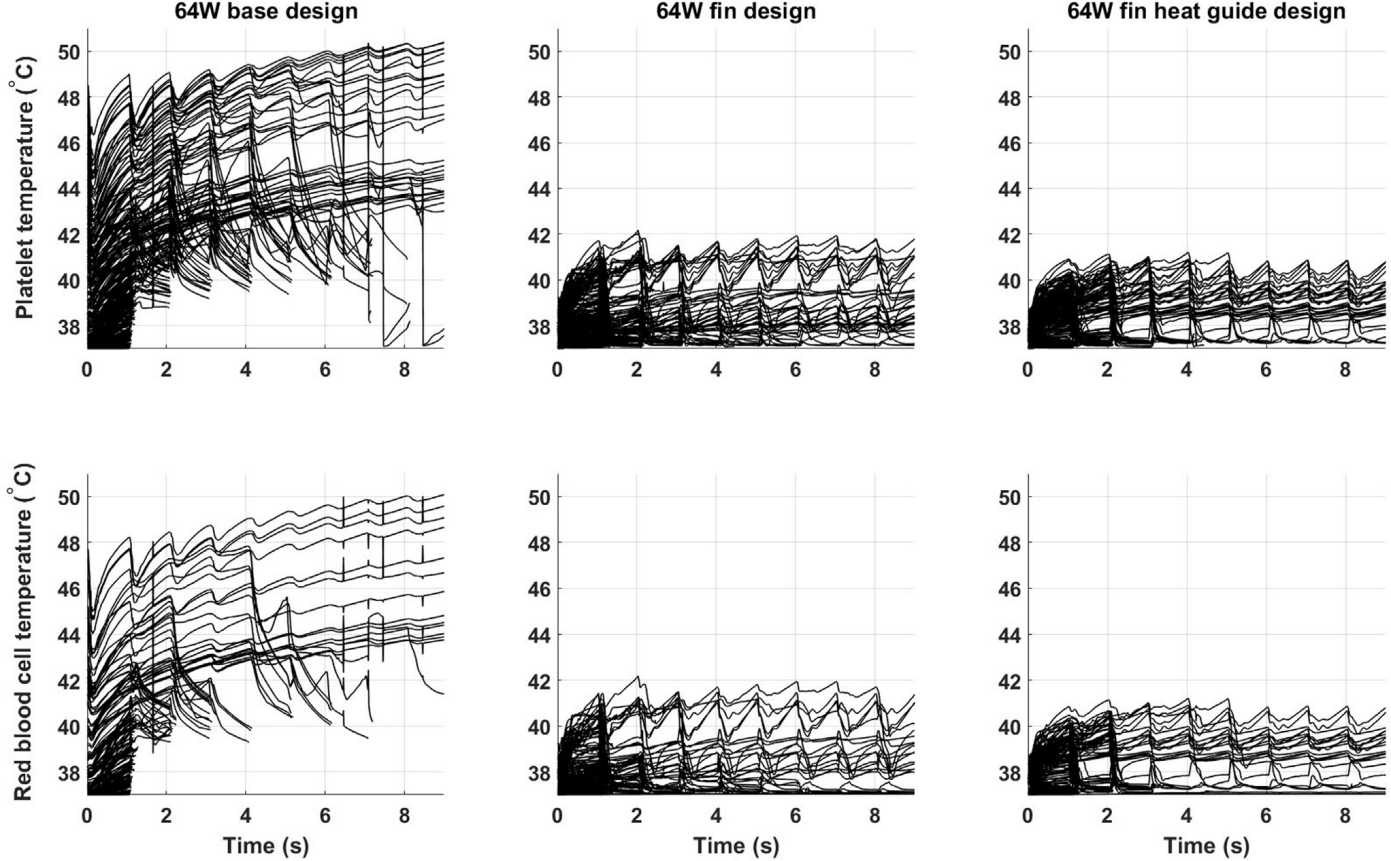
Temperature time-history of 500 platelets and 500 red blood cells flowing through the descending aorta in the 64 W (left) base, (center) fins, and (right) fins with heat guide designs. A significant proportion of the released cells experiences high heat loads for prolonged periods of time in the base design. The addition of the fins and of the heat guide dramatically reduces the maximum temperatures the cells are subjected to. The different initial spatial distribution of platelets and red blood cells leads to a higher fraction of the former being exposed to high heat loads.

Of key importance in the design of a fully implantable heat distribution system are the temperature levels, and exposure times, that cells and proteins are exposed to. To evaluate the heat load cells are subjected to 500 platelets and 500 red blood cells have been tracked for nine cardiac cycles (the first cycle of the 10 simulated was not used due to spurious initialization effects) in the 64 W base, fins, and fins with heat guide design. The temperature time-history is reported in Figure 11. In the base design, a sub-population of cells, both platelets and red blood cells, experiences temperatures around 50°C for several seconds. These temperatures and exposure times might be enough to cause reversible or permanent damage to those cells. A dramatic decrease (>8°C) in temperature is observed, both for platelets and red blood cells, in the fins and fins with heat guide designs compared with the base one. In addition, temperatures are slightly lower in the fins with heat guide design compared to the fins one. This being the result of the more homogeneous heat distribution at the blood-contacting interface achieved with the introduction of the heat guide. The main difference between platelets and red blood cells is the number of cells exposed to the high temperature levels present in the thermal boundary layer, this in turn being due to their initial different spatial distribution.

**Figure 12:**
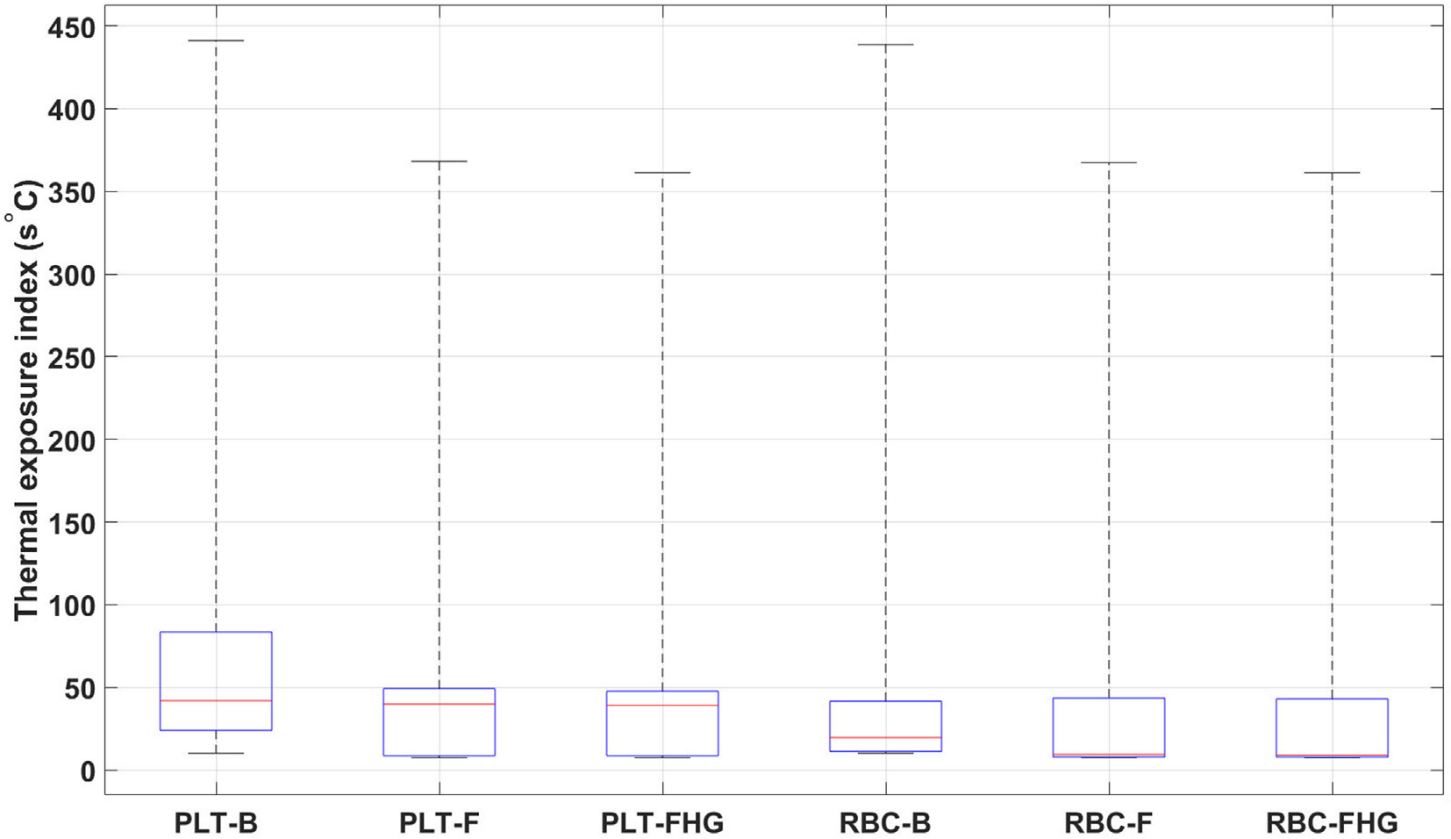
Box plots representing the thermal exposure index distribution for the six analyzed cases. PLT, platelet; RBC, red blood cell; B, base design; F, fins design; FHG, fins with heat guide design. A clear decrease in the maximum TEI and an overall shift of its distribution toward lower values in the fins and fins with heat guide cases, compared to the base cases, is clearly observed. Box plot whiskers’ ends represent the minimum and maximum of all of the data.

From Table 7 and Figure 12, it is possible to observe that the use of fins and fins with heat guide leads to a clear decrease in the maximum TEI value for both cell populations and a clear distribution shift toward lower values. These results show how the more homogeneous heat transfer achieved by the deployment of fins and fins with heat guide can substantially alter the heat load experienced by blood cells.

**Table 7:** Thermal exposure index for the two types of cells investigated in the three 64 W designs.

64 W base model	Max (s·°C)	Mean (s·°C)	Median (s·°C)	Min (s·°C)
Platelets	441.08	81.14	41.93	10.00
Red blood cells	438.78	45.60	19.61	10.00

**64 W fins model**	**Max (s·°C)**	**Mean (s·°C)**	**Median (s·°C)**	**Min (s·°C)**

Platelets	368.11	59.11	39.75	7.40
Red blood cells	367.40	41.78	9.27	7.40

**64 W fins-heat guide model**	**Max (s·°C)**	**Mean (s·°C)**	**Median (s·°C)**	**Min (s·°C)**

Platelets	361.28	54.16	39.04	7.40
Red blood cells	361.28	40.13	8.95	7.40

*The initial spatial distribution, proportional to 1/r^3^ for platelets and uniform for red blood cells, is the main cause of the difference observed between the two type of cells*.

## 4 Discussion

Over five million Americans suffer from heart failure and more than 250,000 die from it every year (Mozaffarian et al., 2016). Mechanical circulatory support devices are a key therapeutic option for end-stage heart failure patients and despite their life-saving role several critical limitations to their widespread use exist (Rose et al., 2001). Among them, the need for an extracorporeal energy source, with the associated percutaneous cables and the risk for infection (Leuck, 2015). The development of a durable, fully implantable energy source would solve one of the major obstacles to the expansion to a wider patient population of mechanical circulatory support therapy. Nuclear energy sources are particularly suitable for this task as they can last years and are sufficiently compact to fit inside the human body. In particular, the thermal energy generated by *α*-emitting materials can be harnessed to generate electricity for powering a heart support device (Huffman et al., 1974; Poirier, 2012; Tchantchaleishvili et al., 2012). To make this approach feasible, the associated waste heat must be locally contained, intracorporeally distributed, and extracorporeally dissipated to the environment. Distributing the waste heat is needed to avoid injuring the tissues surrounding the heat source and to allow its subsequent dissipation through the skin to the environment. Animal tests showed the ability of the blood flow in the descending aorta to remove the waste heat with minimal average blood temperature increase (Huffman et al., 1974). On the other hand, no studies, to the best of authors’ knowledge, have provided a precise assessment of the spatial and temporal temperature and heat flux distributions at the blood–heat exchanger interface and inside the blood flow. This work presents a computational analysis of the coupled solid–fluid heat transfer occurring from the heat source to the blood flow in the descending aorta with different heat exchanger configurations and power levels. Results show that, despite the blood flow in the descending aorta is an ideal heat sink, blood cells, and proteins in the thermal boundary layer are subjected to supra-physiological, and potentially damaging, combinations of temperatures and exposure times if no proper heat exchangers are developed and implanted. In particular, heat exchangers incorporating intraluminal fins and heat guides look as promising designs able to keep blood temperatures at physiologically acceptable levels.

### 4.1 64 and 24 W Base Cases

In both cases, supra-physiological temperatures are present at the blood–heat exchanger interface and inside the blood thermal boundary layer. In particular, the TAH-rated 64 W base model shows time-averaged temperatures exceeding 51°C, while the 24 W base model, aimed at powering an LVAD, presents temperatures approaching 43°C. Therefore, in both cases, temperatures not suitable for endothelial cells survival and potentially damaging for blood cell and protein are reached.

### 4.2 64 W Base, Fins, and Fins with Heat Guide Designs

The 64 W case, being the most demanding in terms of heat distribution, has been chosen as basis for further refinement of the heat exchanger configuration. The addition of the intraluminal fins dramatically improves, spatially and temporally, the heat transfer to the blood so that lower wall and boundary layer temperatures are achieved. In particular, a temperature decrease greater than 8°C is experienced by the blood cells flowing through the descending aorta as reported in Figure 11. The addition of the heat guide further helps improving the spatial heat distribution by guiding the heat toward the far side of the aorta, therefore achieving more spatially homogeneous, and lower, temperature profiles, see Figures 8 and 9. Most importantly, this design is able to transmit 64 W with peak temperatures lower than in the 24 W base design.

### 4.3 Cell and Protein Exposure to Hyperthermia Conditions

While the exposures to supra-physiological temperatures encountered in this work are much shorter than the ones needed to produce significant damage in *in vitro* experiments, see Table 1, *in vivo* conditions, e.g., inflammation, might significantly alter the cellular and protein response by rendering them more susceptible to heat damage. The prolonged exposure to supra-physiological temperatures, in particular in the 64 W base design, might induce temporary or permanent changes in the blood cells and proteins leading to systemic insults. On the other hand, blood components might recover from this suddenly applied heat load as they move through the circulatory system. Moreover, one single passage in the heat-exchanging area might result in sub-critical damage, while multiple passages might lead to critical damage levels and subsequent systemic injury. While cells may become damaged spleen and liver remove damaged red blood cells (Wagner et al., 1962) and platelets (Kaplan and Saba, 1978) from circulation. Under these circumstances, compensatory mechanisms will negate heat-induced damage to blood cells. There is a pressing need for more experimental data on the effect of internal heat loads on tissues, cells, and the systemic response.

### 4.4 Future Work and Limitations

As any analysis of complex biological systems, this work highlights new future research directions. Also, the work has been based on several assumptions and limitations; the most important are addressed below.

#### 4.4.1 Perfect Insulation

The present models consider a perfect insulation of the casing containing the heat source and of the artery walls. In reality, a fraction of the generated heat will be transmitted to the surrounding tissues, through the casing and the arterial tissue. This will lead to neovascularization of the affected areas as a mean to remove the extra heat load allowing for the “leaked” heat to be distributed throughout the entire body via the blood circulation. Despite short-term tissue adaptation has been reported (Davies et al., 1994), with longer term adaptation resulting in formation of fibrous capsules, which lower tissue temperature through formation of hypervascular networks (Seese et al., 1998), no data exist on the long-term effect of intracorporeal heat on tissues. Further modeling efforts should address this aspect by considering the heat transfer to the surrounding tissues and their, albeit approximated, neovascularization response to evaluate its effect on the heat load in the descending aorta.

#### 4.4.2 Idealized Heat Guide Geometry

This work considered an idealized heat guide geometry. More advanced designs are likely to deliver a much higher heat fraction to the far side of the blood channel, thus further decreasing peak temperatures.

#### 4.4.3 Fins Biocompatibility

Current LVADs and TAHs present large areas of foreign materials, e.g., titanium, in contact with blood. In addition, they introduce non-physiological flow fields characterized by high shear stresses thought of promoting abnormal clotting and cell and protein damage (Eckman and John, 2012; Blitz, 2014). In this work, intraluminal fins have been introduced to enhance the heat transfer capabilities with the aim of reducing wall and boundary layer temperatures. Due to limitation in the geometric modeling capabilities of the employed software fins were created with flat leading and trailing faces. Fins with optimized shapes reducing shear stresses and recirculation regions will likely avoid, or drastically reduce, the potential for thrombus formation. It is important to note that the modeled fins still present a much lower surface area in contact with blood that the one in LVADs and TAHs and also lower shear stress values. Future work should carefully address the design of such fins to minimize their thrombogenic footprint.

#### 4.4.4 Weighted TEI vs Linear TEI

In the TEI calculation, no distinction has been made with regard to temperature or time exposure values. In reality, different temperatures and exposure times, e.g., above a critical level, might contribute differently to the TEI.

#### 4.4.5 Computational Mesh

The computational meshes employed in this work are fine enough to achieve mesh convergence and, therefore, they allow the capturing of the underlying physics. More refined meshes might be needed to capture smaller scale flow features and their associate heat transfer phenomena. The main limitation for the use of finer meshes is the computational cost associated with them. Future work should be directed toward more extensively exploiting parallel computing capabilities so as to be able to simulate several cardiac cycles with a higher spatial and temporal resolution.

## Author Contributions

JB formulated the scientific problem, performed all the computations and data analysis, and wrote the manuscript. AP assisted in formulating the clinical and biological aspect of the problem and critically revised the manuscript. PGS contributed in the heat transfer analysis and in revising the manuscript.

## Conflict of Interest Statement

The authors declare that the research was conducted in the absence of any commercial or financial relationships that could be construed as a potential conflict of interest. The handling editor declared a shared affiliation, though no other collaboration, with several of the authors AP, JB, and the handling editor states that the process met the standards of a fair and objective review.

## Footnotes

^1^*Leviticus Cardio CET VAD System* (2017). Available at: http://leviticuscardio.com/default.asp

## References

[B1] Aarts P. A. van den Broek S. A. Prins G. W. Kuiken G. D. Sixma J. J. Heethaar R. M. (1988). Blood platelets are concentrated near the wall and red blood cells, in the center in flowing blood. Arteriosclerosis 8, 819–824. 10.1161/01.ATV.8.6.819 3196226

[B2] Alastruey J. Xiao N. Fok H. Schaeffter T. Figueroa C. A. (2016). On the impact of modelling assumptions in multi-scale, subject-specific models of aortic haemodynamics. J. R. Soc. Interface 13, 20160073. 10.1098/rsif.2016.0073 27307511 PMC4938079

[B3] Biasetti J. Gasser T. C. Auer M. Hedin U. Labruto F. (2010). Hemodynamics of the normal aorta compared to fusiform and saccular abdominal aortic aneurysms with emphasis on a potential thrombus formation mechanism. Ann. Biomed. Eng. 38, 380–390. 10.1007/s10439-009-9843-6 19936925

[B4] Biasetti J. Hussain F. Gasser T. C. (2011). Blood flow and coherent vortices in the normal and aneurysmatic aortas: a fluid dynamical approach to intra-luminal thrombus formation. J. R. Soc. Interface 8, 1449–1461. 10.1098/rsif.2011.0041 21471188 PMC3163425

[B5] Blitz A. (2014). Pump thrombosis—a riddle wrapped in a mystery inside an enigma Keynote Lecture Series. Ann. Cardiothorac. Surg. 3, 450–471. 10.3978/j.issn.2225-319X.2014.09.10 25452905 PMC4229470

[B6] Bouchama A. Bridey F. Hammami M. M. Lacombe C. Al-Shail E. Al-Ohali Y. (1996). Activation of coagulation and fibrinolysis in heatstroke. Thromb. Haemost. 76, 909–915. 8972010

[B7] Bouchama A. Knochel J. P. (2002). Heat stroke. N. Engl. J. Med. 346, 1978–1988. 10.1056/NEJMra011089 12075060

[B8] Choi J. W. Pai S. H. (2002). Changes in hematologic parameters induced by thermal treatment of human blood. Ann. Clin. Lab. Sci. 32, 393–398. 12458892

[B9] Davies C. R. Fukumura F. Fukamachi K. Muramoto K. Himley S. C. Massiello A. (1994). Adaptation of tissue to a chronic heat load. ASAIO J. 40, M514–M517. 10.1097/00002480-199407000-00053 8555569

[B10] Diez-Silva M. Dao M. Han J. Lim C.-T. Suresh S. (2010). Shape and biomechanical characteristics of human red blood cells in health and disease. MRS Bull. 35, 382–388. 10.1557/mrs2010.571 21151848 PMC2998922

[B11] Eckman P. M. John R. (2012). Bleeding and thrombosis in patients with continuous-flow ventricular assist devices. Circulation 125, 3038–3047. 10.1161/CIRCULATIONAHA.111.040246 22711669

[B12] Emoto H. Harasaki H. Fujimoto L. K. Navarro R. R. White M. Whalen R. (1988). Systemic and local effects of heat dissipation in the thermally powered LVAS. ASAIO Trans., 34, 361–366. 2461726

[B13] Etulain J. Lapponi M. J. Patrucchi S. J. Romaniuk M. A. Benzadón R. Klement G. L. (2011). Hyperthermia inhibits platelet hemostatic functions and selectively regulates the release of alpha-granule proteins. J. Thromb. Haemost. 9, 1562–1571. 10.1111/j.1538-7836.2011.04394.x 21649851 PMC3155010

[B14] Gershfeld N. L. Murayama M. (1988). Thermal instability of red blood cell membrane bilayers: temperature dependence of hemolysis. J. Membr. Biol. 101, 67–72. 10.1007/BF01872821 3367362

[B15] Huffman F. N. Ph D. Hagen K. G. Whalen R. L. Fuqua J. M. Norman J. C. (1974). Intracorporeal heat dissipation firom a radioisotope-powered artificial heart. Bull. Tex. Heart Inst. 1, 343–368. PMC287500 15215968

[B16] Jamiolkowski M. A. Pedersen D. D. Wu W. T. Antaki J. F. Wagner W. R. (2016). Visualization and analysis of biomaterial-centered thrombus formation within a defined crevice under flow. Biomaterials 96, 72–83. 10.1016/j.biomaterials.2016.04.022 27156141 PMC4982661

[B17] Jamiolkowski M. A. Woolley J. R. Kameneva M. V. Antaki J. F. Wagner W. R. (2015). Real time visualization and characterization of platelet deposition under flow onto clinically relevant opaque surfaces. J. Biomed. Mater. Res. A 103, 1303–1311. 10.1002/jbm.a.35202 24753320 PMC4203704

[B18] Kaplan J. E. Saba T. M. (1978). Platelet removal from the circulation by the liver and spleen. Am. J. Physiol. 235, H314–H320. 696842 10.1152/ajpheart.1978.235.3.H314

[B19] Knecht O. Bosshard R. Kolar J. W. Starck C. T. (2014). “Optimization of trancutaneous energy transfer coils for high power medical applications,” in Proceedings of the 15th IEEE Workshop on Control and Modeling for Power Electronics (COMPEL 2014) (Santander, Spain: Compel).

[B20] Leuck A.-M. (2015). Left ventricular assist device driveline infections: recent advances and future goals. J. Thorac. Dis. 7, 2151–2157. 10.3978/j.issn.2072-1439.2015.11.06 26793335 PMC4703684

[B21] MacIver J. Ross H. J. (2012). Quality of life and left ventricular assist device support. Circulation 126, 866–874. 10.1161/CIRCULATIONAHA.111.040279 22891167

[B22] Mezzano D. Hwang K. L. Catalano P. Aster R. H. (1981). Evidence that platelet buoyant density, but not size, correlates with platelet age in man. Am. J. Hematol. 11, 61–76. 10.1002/ajh.2830110108 7270546

[B23] Mozaffarian D. Benjamin E. J. Go A. S. Arnett D. K. Blaha M. J. Cushman M. (2016). Heart Disease and Stroke Statistics-2016 Update a Report from the American Heart Association. Circulation. 133(4):e38–e48. 10.1161/CIR.0000000000000350 26673558

[B24] Pasha R. Benavides M. Kottke-Marchant K. Harasaki H. (1995). Reduced Expression of Platelet Surface Glycoprotein Receptor IIb/IIIa at Hyperthermic Temperatures. 7564273

[B25] Poirier V. (2012). Will we see nuclear-powered ventricular assist devices? ASAIO J. 58, 546–547. 10.1097/MAT.0b013e31826e3ee6 23103695

[B26] Polanowska-Grabowska R. Raha S. Gear A. R. L. (1992). Adhesion efficiency, platelet density and size. Br. J. Haematol. 82, 715–720. 10.1111/j.1365-2141.1992.tb06949.x 1282829

[B27] Rose A. E. Gelijns C. A. Moskowitz A. Heitjan F. D. Stevenson W. L. Dembitsky W. (2001). Long-term use of a left ventricular assist device for end-stage heart failure. N. Engl. J. Med. 345, 1435–1443. 10.1056/NEJMoa012175 11794191

[B28] Seese T. M. Harasaki H. Saidel G. M. Davies C. R. (1998). Characterization of tissue morphology, angiogenesis, and temperature in the adaptive response of muscle tissue to chronic heating. Lab. Invest. 78, 1553–1562. 10.3109/00952990.2011.553977 9881955

[B29] Sohal R. S. Chien Sun S. Colcolough H. L. Burch G. E. (1968). Heat stroke: an electron microscopic study of endothelial cell damage and disseminated intravascular coagulation. Arch. Intern. Med. 122, 43–47. 10.1001/archinte.1968.00300060045008 5659376

[B30] Tchantchaleishvili V. Bush B. S. Swartz M. F. Day S. W. Massey H. T. (2012). Plutonium-238. ASAIO J. 58, 550–553. 10.1097/MAT.0b013e31826a9204 23085941

[B31] Utoh J. Harasaki H. (1992). Effects of temperature on phagocytosis of human and calf polymorphonuclear leukocytes. Artif. Organs 16, 377–381. 10.1111/j.1525-1594.1992.tb00535.x 10078278

[B32] Utoh J. Zajkowski-Brown J. E. Harasaki H. (1992). Effects of heat on fragility and morphology of human and calf erythrocytes. J. Invest. Surg. 5, 305–313. 10.3109/08941939209012448 1472484

[B33] Wagner H. N. J. Razzak M. A. Gaertner R. A. Caine W. P. J. Feagin O. T. (1962). Removal of erythrocytes from the circulation. Arch. Intern. Med. 110, 90–97. 10.1001/archinte.1962.03620190092014 14004365

[B34] Wang J. X. Smith J. R. Bonde P. (2014). Energy transmission and power sources for mechanical circulatory support devices to achieve total implantability. Ann. Thorac. Surg. 97, 1467–1474. 10.1016/j.athoracsur.2013.10.107 24530103

[B35] Whalen R. L. Jeffery D. L. Asimacopoulos P. J. Norman J. C. (1974). Chronic intracorporeal heat studies in calves. Trans. Am. Soc. Artif. Intern. Organs 20, 509–515. 4450302

